# Accelerated Spatial Fibrin Growth and Impaired Contraction of Blood Clots in Patients with Rheumatoid Arthritis

**DOI:** 10.3390/ijms21249434

**Published:** 2020-12-11

**Authors:** Alina D. Peshkova, Tatiana A. Evdokimova, Timur B. Sibgatullin, Fazoil I. Ataullakhanov, Rustem I. Litvinov, John W. Weisel

**Affiliations:** 1 Institute of Fundamental Medicine and Biology, Kazan Federal University, Kazan 420008, Russia; alinapeshkova26@gmail.com (A.D.P.); rustempa@gmail.com (T.A.E.); litvinov@pennmedicine.upenn.edu (R.I.L.); 2 Department of Rheumatology, University Hospital, Kazan Federal University, Kazan 420008, Russia; nellalit.54@gmail.com; 3 Center for Theoretical Problems of Physico-Chemical Pharmacology, Russian Academy of Sciences, Moscow 119991, Russia; ataullakhanov.fazly@gmail.com; 4 Department of Cell and Developmental Biology, University of Pennsylvania School of Medicine, Philadelphia, PA 19104, USA

**Keywords:** rheumatoid arthritis, blood coagulation, hypercoagulability, contraction of blood clots

## Abstract

Rheumatoid arthritis (RA) is an autoimmune disease associated with thrombotic complications. To elucidate pathogenic mechanisms, hemostatic disorders in RA were correlated with other laboratory and clinical manifestations. Hemostasis was assessed using relatively new complementary tests, the spatial growth of a plasma clot (Thrombodynamics assay), and contraction of whole blood clots. Platelet functionality was assessed with flow cytometry that quantified the expression of P-selectin and the fibrinogen-binding capacity of platelets before and after activation with a thrombin receptor-activating peptide. Parameters of fibrin clot growth and the kinetics of contraction of blood clots were significantly altered in patients with RA compared to the control group. In Thrombodynamics measurements, an increase in the clot growth rate, size, and optical density of plasma clots altogether indicated chronic hypercoagulability. The rate and extent of blood clot contraction in patients with RA was significantly reduced and associated with platelet dysfunction revealed by an impaired response to activation. Changes in the parameters of clot growth and contraction correlated with the laboratory signs of systemic inflammation, including hyperfibrinogenemia. These results confirm the pathogenic role of hemostatic disorders in RA and support the validity of fibrin clot growth and the blood clot contraction assay as indicators of a (pro)thrombotic state.

## 1. Introduction

Rheumatoid arthritis (RA) is a chronic autoimmune disease characterized by inflammation associated with pain, edema, and deformation in joints, resulting in immobility and disability [1]. The prevalence of RA in the general population varies from 0.25% to 2.5% [2,3,4]. Most RA studies focus on combating joint damage; however, RA commonly has severe extra-articular manifestations, including thrombotic complications, such as myocardial infarction, ischemic stroke, and venous thromboembolism [5,6,7].

Although RA is not a direct cause of thrombosis, chronic inflammation in RA together with prothrombotic changes in blood composition and endothelial dysfunction lead to the development of atherothrombosis [8]. The development of thrombosis in RA is promoted by a high frequency of lesions of the peripheral arteries and veins, which comprises 19.6% and 7.2% of patients, respectively [9]. Inflammation of a joint is accompanied by production and secretion into the blood of cytokines, which induce a prothrombotic state by activating endothelium, resulting in expression of tissue factor, as well as inhibition of the fibrinolysis and protein C pathways [5,10]. Important prothrombotic factors in RA are activated platelets [11] and platelet-derived microvesicles [12]. Thrombocytosis and an increase in the average platelet volume in patients with RA indicate a stimulation of thrombocytopoiesis, probably under the influence of chronic inflammation mediators [11]. The key cytokine in this process is interleukin-6 (IL-6) [13,14], which enhances the formation of platelets from megakaryocytes; however, the mechanism of this effect in RA is not fully understood [15]. A high level of platelet microvesicles in the blood was found in experimental RA [16,17], an increase in the level of platelet microvesicles in blood plasma [18,19] and synovial microvesicles is also observed in patients with RA [20]. In the blood of patients with RA, elevated levels of fibrinogen [21,22,23], tissue-type plasminogen activator [24,25], tissue factor [26], plasminogen activator inhibitor-1 [25,27], and D-dimer [22,26,28] were found, which together indicate hypercoagulability and a pre-thrombotic condition [6,29]. All of the above provides mechanistic evidence that RA is a risk factor for thrombotic complications that lead to relatively high rates of premature mortality from cardiovascular diseases in patients with early seropositive arthritis [9].

Despite the importance of the prognosis and early diagnosis of imminent or ongoing thrombosis, complicating different types of pathology, traditional techniques for laboratory assessment of hemostasis are aimed mostly at elucidating defects of blood clotting and are not as effective in the evaluation of the thrombotic potential, including that in rheumatic diseases. To improve this methodological flaw, a new integral hemostatic test called the Thrombodynamics assay has been recently introduced [30]. This assay is sensitive to both hypo- and hypercoagulability, capturing subtle hemostatic disorders, including those underlying (pro)thrombotic states. The test is based on local activation of the coagulation system in a platelet-free blood plasma sample from one side by surface-attached tissue factor, mimicking the procoagulant surface of a damaged vessel wall. The surface-induced activation of the clotting cascade is followed by spatiotemporal fibrin clot growth, which is tracked optically in real-time [31,32].

After the formation of a blood clot, either in vitro or in vivo, it undergoes volumetric shrinkage called clot contraction or retraction. Clot contraction is driven by activated platelets; hence this process can be used as a test to evaluate platelet count and functionality. In addition to platelets, blood clot contraction is modulated by pathological changes in the cellular and molecular composition of the blood [33]. Therefore, the kinetics of clot contraction may provide important integrated information on the overall hemostatic/thrombotic potential. Clinical studies in patients with (pro)thrombotic conditions, such as ischemic stroke [34], venous thromboembolism [35], systemic lupus erythematosus [36], and miscarriage [37] have shown that clot contraction is significantly reduced due to platelet dysfunction as a result of their chronic activation followed by energetic exhaustion and refractoriness to biochemical stimulation [34,36,37,38]. Hypothetically, similar processes may take place in the blood of patients with RA. Studying clot contraction in the blood of these patients may provide additional information about the role of hemostatic disorders in the pathogenesis and extra-articular clinical manifestations of RA.

The aim of this work was to study the status of hemostasis in patients with RA using a combination of two relatively new complementary laboratory assays, namely the Thrombodynamics assay and the kinetics of blood clot contraction. The results of these tests are correlated with other laboratory test and clinical characteristics that indicate systemic inflammation and the severity of the disease.

## 2. Results

### 2.1. Hypercoagulability in Patients with RA Revealed by the Thrombodynamics Assay

**Figure 1:**
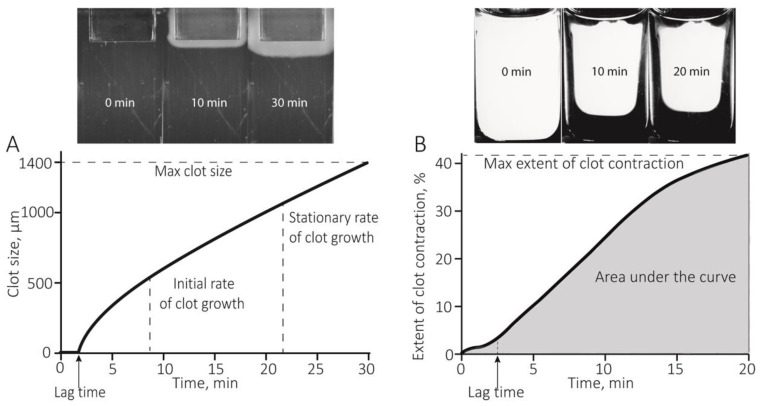
Results of the optical system used to quantify the spatial growth of fibrin clots (Thrombodynamics) (**A**) and the kinetics of contraction (retraction) of blood clots as a function of time (**B**). At the top are images of the cuvettes for clot growth (**A**) and clot contraction (**B**) and below are graphs of the output of these assays. For details see Materials and Methods (Section 4.3 and Section 4.4).

**Figure 2:**
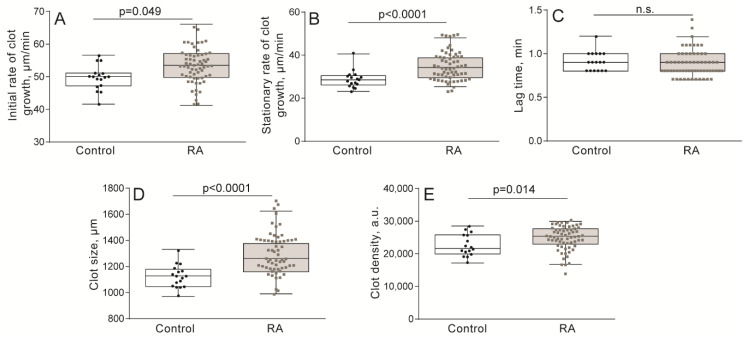
Parameters to quantify fibrin clot growth from Thrombodynamics measurements in patients with rheumatoid arthritis (RA) (*n* = 60) compared with the control group (*n* = 50). The initial growth rate of the clot (**A**), stationary growth rate of the clot (**B**), lag time (**C**), clot size (**D**), and clot density (**E**). The results are presented as the median and intervals between 25th and 75th, as well as between 5th and 95th percentiles (Mann-Whitney U test). n.s.—not significant.

The Thrombodynamics assay is based on the optical registration of the spatial growth of a fibrin clot in blood plasma (Figure 1A). Thrombodynamics assays in RA patients compared with the control group revealed significantly higher median initial and stationary clot growth rates, as well as larger clot size and optical density (Figure 2 and Supplementary Materials Table S1). In addition, in 13 (22%) patients, in 21–29 min after the initiation of clotting by immobilized tissue factor, the formation of “spontaneous” clots all over the plasma volume was observed; these clots were not related to the tissue factor-induced local clotting, indicating strong hypercoagulability and thrombin generation in bulk following plasma recalcification.

Correlation analysis of the parameters of the Thrombodynamics assay and other hemostatic and hematologic tests (Table S2A) revealed the following significant associations. Expectedly, there was a strong correlation between some parameters of the Thrombodynamics assay, such that the initial (*Vi*) and stationary (*Vst*) rates of clot growth correlated directly with each other (r = 0.587) and were linked tightly to the clot size (r = 0.776 and r = 0.947, respectively). The clot optical density (*D*) was directly and expectedly associated with the fibrinogen levels (r = 0.535), while there was a modest inverse correlation of the fibrinogen levels and *Vst* (r = −0.236). Notably, *Vi* and *Vst* both directly correlated, moderately but significantly, with the extent of clot contraction (r = 0.233 and r = 0.246, respectively) and *Vi* was inversely proportional to the aPTT (r = −0.384), indicating common underlying mechanisms related to enhanced thrombin generation.

The correlation analysis revealed potential mechanistic links between the parameters of the Thrombodynamics assay and laboratory signs of inflammation. Namely, a faster erythrocyte sedimentation rate (ESR) was associated with slower *Vst* (r = −0.218) and higher *D* (r = 0.321), both parameters of the Thrombodynamics assay following the same trend as with fibrinogen, which likely reflects hyperfibrinogenemia associated with an acute phase reaction. The lag time was prolonged at the higher anti-DNA antibody titers (r = 0.435) and *Vi* decreased with an increase of anti-cardiolipin antibody level (r = −0.327), both trends reflecting the production of autoantibodies, including anti-phospholipid antibodies, which inhibit thrombin generation in vitro, but promote thrombosis in vivo [39]. Significantly, the time of spontaneous clot formation was substantially shorter with higher monocyte counts (r = −0.654), confirming indirectly that the spontaneous clots are induced by high thrombin activity unrelated to the tissue factor immobilized on the plastic insert in the instrument used to initiate clotting. Altogether, these results validate the Thrombodynamics assay as an integral method for studying hemostasis that is sensitive to systemic inflammation and pathological alteration of blood composition, leading to pronounced hypercoagulability in RA patients.

### 2.2. Impaired Kinetics of Clot Contraction in the Blood of Patients with RA

**Figure 3:**
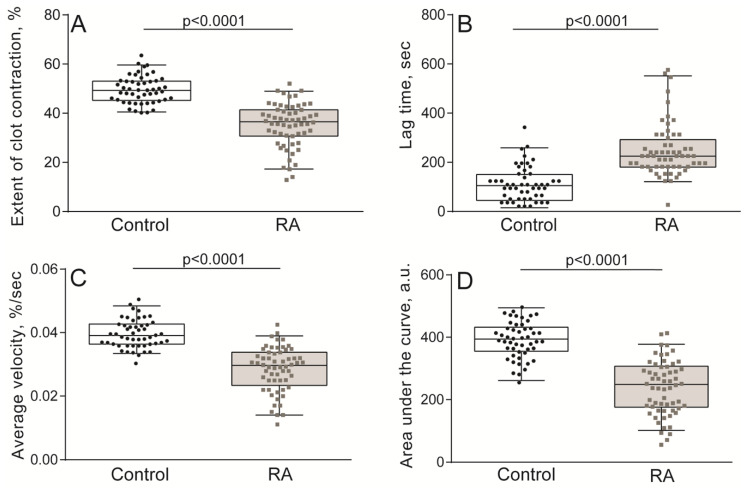
Quantification of blood clot contraction in RA patients (*n* = 60) compared with those of the control group (*n* = 50). The extent of clot contraction (**A**), lag time of contraction (**B**), average velocity of contraction (**C**), and area under the curve (work performed by platelets in contraction) (**D**). The results are presented as the median and intervals between the 25th and 75th, as well as between the 5th and 95th percentiles (Mann-Whitney U test).

**Figure 4:**
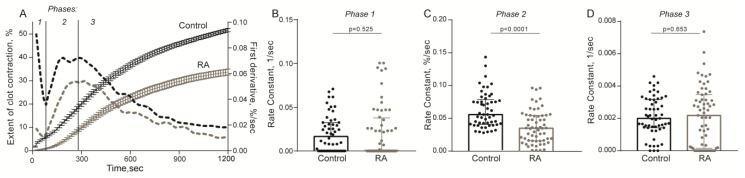
Comparative phase analysis of the averaged kinetic curves of clot contraction for RA patients (*n* = 63) and control group (*n* = 50). For the kinetic curves (**A**), transitions between phases 1, 2, and 3 of clot contraction were determined by finding local maxima and minima within the instantaneous first derivatives (dashed lines). The curves were fit using a piecewise function, and the rate constants of each phase were determined [33]. Rate constants of phase 1 (**B**), phase 2 (**C**), and phase 3 (**D**) are shown for RA patients and the control group. Data are presented as the median and interquartile ranges (25th and 75th percentiles) (Mann-Whitney U test).

The kinetics of the contraction of blood clots was recorded in vitro using different functionalities of the same Thrombodynamics Analyzer System (Figure 1B). Compared to the healthy control subjects, patients with RA had significantly suppressed contraction of blood clots, reflected by the smaller median extent of contraction, the prolonged lag period, the reduced average velocity, and lesser area under the kinetic curves (Figure 3, Table S3). Clot contraction is known to occur in three phases: initiation of contraction (phase 1), linear contraction (phase 2), and mechanical stabilization (phase 3) [33]. Regression analysis conducted on the averaged kinetic curves and their first derivatives (Figure 4A and Table S4) revealed that in RA patients the rate constant of phase 2 was significantly reduced compared to that of the healthy subjects (Figure 4C), indicating that clot contraction was suppressed in RA due to impairment of the active biomechanical contractile mechanisms that lead to compaction of the clots. There were no significant differences in the rates of phases 1 and 3 between healthy controls and RA patients (Figure 4B,D), suggesting that there were no defects in the contraction initiation and in the mechanical stabilization of contracted clots in the blood of RA patients.

To identify possible pathogenic mechanisms of the impaired clot contraction in the blood of RA patients, the parameters of contraction kinetics were compared with the results of other laboratory tests. The substantial and meaningful results of the correlation analysis are shown in Table S2B. First, the parameters of clot contraction showed a remarkable inter-correlation, indicating their mechanistic connectivity. The final extent of clot contraction correlated strongly and directly with the area under the curve (r = 0.843) and average velocity (r = 0.922), altogether characterizing the contractile power of platelets, which is a measure of the amount of work done in a given amount of time. Next, there was an inverse relationship between the lag time and the final extent of contraction (r = −0.484), area under the kinetic curve or the mechanical work done on clot shrinkage (r = −0.645), and the average velocity of contraction (r = −0.482), together indicating the functional importance of the initiation of clot contraction for its further kinetics and the ultimate degree of compaction. Further and expectedly, platelet count correlated with the extent of clot contraction (r = 0.321), area under the kinetic curve (r = 0.320), and median velocity (r = 0.369); however, the moderate degree of this direct correlation suggests that clot contraction can be modulated by factors other than platelet counts. In line with this notion, the extent of clot contraction correlated inversely with the RBC count (r = −0.295) and hematocrit (r = −0.255), confirming our earlier study showing suppression of clot contraction by RBCs [33].

Remarkably, a relatively higher extent of clot contraction was associated with the more pronounced laboratory signs of inflammation, namely with a positive rheumatoid factor test and ESR. In seropositive RA patients (*n* = 50), the median extent of clot contraction was 37 (IQR: 32; 43), while in seronegative RA patients (*n* = 10), it was 32 (22; 39; *p* = 0.050). In patients with ESR > 40 mm/h (*n* = 25), the median extent of clot contraction was 39 (IQR: 35; 44), while in patients with ESR < 40 mm/h (*n* = 35), the median extent of clot contraction was 35 (27; 39; *p* = 0.006). Although the extent of contraction in all the RA patients was below the normal values (<41%) [40], the relative enhancement of clot contraction associated with marked inflammation agrees with immune platelet activation [41,42].

The aggregate of data indicates the profound impairment of clot contraction in the blood of RA patients and links the altered clot contraction to pathological changes in blood composition, including platelet and RBC counts, as well as signs of systemic inflammation.

**Figure 5:**
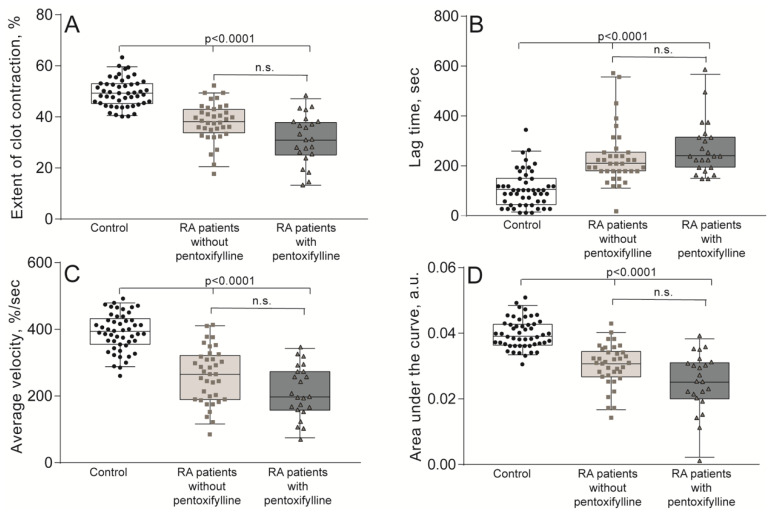
Quantification of contraction of blood clots in patients with RA, not taking (*n* = 37) and taking (*n* = 23) pentoxifylline, compared with the control group (*n* = 50). The extent of clot contraction (**A**), lag time of contraction (**B**), average velocity of contraction (**C**), and area under the curve (work performed by platelets in contraction) (**D**). The results are presented as the median and intervals between 25th and 75th, as well as between 5th and 95th percentiles (Kruskal-Wallis test and Dunn’s multiple post-hoc test). n.s.—not significant.

In addition to natural pathogenic mechanisms that affect hemostasis in chronic autoimmune diseases, such as RA, there is a chance that the observed changes in laboratory tests could be influenced by medications, namely by non-steroidal anti-inflammatory drugs (NSAIDs) and pentoxifylline, which is known to have anti-platelet activity. To determine the potential effect of pentoxifylline on clot contraction, we analyzed the parameters of clot contraction in the RA patients taking (38%) and not taking this drug at the time of examination. There were no significant differences between the clinical subgroups, neither in the median extent and velocity of clot contraction, nor the lag time and area under the kinetic curves (Figure 5). Similarly, we compared the parameters of clot contraction in patients with RA taking (58%) and not taking NSAIDs. Again, no significant changes in the kinetics of clot contraction between the two subgroups were revealed (Table S5). These results rule out possible effects of NSAID and pentoxifylline on clot contraction and support the conclusion that the impaired clot contraction revealed in RA patients is associated with the pathogenesis of the disease.

### 2.3. Platelet Dysfunction in RA Patients

As platelets are critical for the contractile force generated during clot contraction, we studied directly platelet functionality in RA patients, using flow cytometry to evaluate the status of unstimulated platelets and their responsiveness to activation by TRAP, a thrombin mimetic peptide acting on PAR1 receptors. The platelet background state and reactivity were assessed by surface expression of P-selectin as a sign of α-granule secretion and by the ability to bind fibrinogen as a measure of the integrin αIIbβ3 activation.

In unstimulated platelets isolated from the blood of RA patients and control healthy subjects, there was a slight insignificant difference in the background activation of platelets, estimated by the levels of P-selectin expression and αIIbβ3 activation (Table 1 and Figure S1). However, unlike resting non-activated platelets, after stimulation with TRAP, platelets from the blood of RA patients had significantly lower levels of P-selectin expression and fibrinogen-binding capacity both at 3 and 10 min after the TRAP-induced stimulation (Table 1 and Figure S1). The median levels of P-selectin expression and αIIbβ3 activation were 1.3–1.8-fold smaller in the RA platelets versus control after treatment with TRAP.

Collectively, these results indicate that in the blood of RA patients, platelets can be spontaneously activated to a certain extent but more importantly, they have a remarkably reduced responsiveness to a physiological thrombin-like activating stimulus, a phenomenon also known as partial refractoriness. The reduced exposure of P-selectin and fibrinogen-binding activity of platelets in the blood of RA patients indicates a substantially decreased overall activation potential or platelet dysfunction, which likely accounts for the impaired thrombin-induced contraction of blood clots in RA (Figure 3 and Figure 4, Tables S3 and S4).

## 3. Discussion

Many studies have shown that the frequency of arterial and venous thrombosis increases in RA, although the causes and mechanisms of these complications are not completely clear [9,43,44]. To better understand this problem, we have studied hemostatic changes in patients with RA using two relatively new laboratory tests, namely the Thrombodynamics assay and the contraction of blood clots, which have had limited clinical applications and can provide new information on the cellular and molecular mechanisms of thrombotic complications in RA.

The main findings are that in the blood of patients with RA, compared with healthy subjects, pronounced hypercoagulability was observed in combination with a marked decrease in the ability of blood clots to contract. Hypercoagulability in RA was previously detected using ROTEM/TEG [45] and was attributed to endothelial dysfunction associated with the expression of tissue factor, as well as inhibition of the fibrinolytic system, which were combined with an increase in blood levels of inflammatory reactants, such as C-reactive protein and fibrinogen [25,29,46]. Reduced contraction of blood clots in RA has not been described previously, but it was revealed in some other (pro)thrombotic conditions [34,37,38], including autoimmune pathologies, such as systemic lupus erythematosus [36] and asthma [47]. As shrinkage of blood clots is driven by platelets, the suppressed contraction suggests severe platelet dysfunction in RA as an underappreciated pathogenic mechanism.

The role of platelets in the pathogenesis of RA has been suggested in isolated studies, primarily in association with increased production and effects of inflammatory cytokines [5,48]. An increased concentration of platelets in the blood of RA patients was directly related to the signs of inflammation, such as elevated ESR, C-reactive protein and other acute-phase proteins, the appearance of rheumatoid factor, leukocytosis, etc. [48,49]. Chronic platelet activation was accompanied by the expression of phosphatidylserine, the formation of a procoagulant surface, and the release of a large number of platelet microvesicles, which also have procoagulant activity and high prothrombotic potential [50,51]. In line with the observations that platelets in RA undergo stimulation as a result of systemic inflammation, we found relative rises in the degree and speed of contraction in patients with ESR > 40 mm/h and seropositive RA patients. The most likely mechanism for platelet activation in RA is the direct effect of circulating immune complexes mediated through FcγRIIA receptors [52], including immune complexes formed by antibodies against the cyclic citrullinated peptide (ACCP), which are specific for RA [41,52].

Paradoxically, despite the relative thrombocytosis and immune activation of platelets, the most prevalent observation is impaired contraction of blood clots, indicative of profound platelet dysfunction. After excluding the possibility that reduced platelet contractility might be due to antiplatelet activity of drugs used to treat RA (Figure 5 and Table S5), we explored platelet functionality before and after stimulation with a thrombin mimetic peptide capable of activating platelets via PAR1 receptors [53]. Our results have demonstrated clearly that platelets from the blood of RA patients are dysfunctional and have a reduced ability to respond to the stimulus adequately by promoting α-granule secretion (assessed by P-selectin expression) and exposure of active integrin αIIbβ3 (assessed by the ability to bind fibrinogen) (Table 1 and Figure S1). The most plausible explanation for the platelet dysfunction and reduced contractility in RA is continuous immune platelet activation in the bloodstream induced by immune complexes [54] and by systemic thrombinemia due to cellular activation followed by expression of tissue factor and procoagulant phospholipids on cells and cellular microvesicles [41,42,55]. As the pathological process and systemic inflammation develop, platelet functionality is compromised due to energy depletion, exhaustion, and secondary refractoriness [51], which explains the suppression of contraction of blood clots in RA. Similar mechanisms of impaired clot contraction due to platelet exhaustion were described in ischemic stroke [34], DVT [35], SLE [36], and miscarriages [37].

In addition to the altered platelet functionality, pathological changes in the composition of blood could contribute to variations in clot contraction. One of the most likely reasons for reduced clot contraction is the high concentration of fibrinogen in the blood of patients with RA, reflected by the high optical density of clots in the Thrombodynamics assay (Figure 2, Table S1). Hyperfibinogenemia has been shown to be associated with a reduced extent and rate of clot contraction, both in vitro [33] and in vivo [56,57]. High concentrations of fibrin(ogen) increase the density of the fibrin network and change its structure [58], resulting in increased stiffness of clots and reduced permeability of the fibrin network for fibrinolytic enzymes, which further increase the prothrombotic potential [46].

The results of this study provide additional laboratory evidence for coagulopathies in RA and elucidate the mechanisms underlying a higher incidence of thrombotic complications in RA patients. This work provides new means to reveal RA patients at a high risk of thrombotic complications and emphasizes the importance of timely thromboprophylaxis with anticoagulants and antiplatelet drugs in this patient category.

## 4. Materials and Methods

### 4.1. Patients, Healthy Subjects, and Inclusion and Exclusion Criteria

The main group consisted of 60 consecutive patients with RA 22–78 years old (on average 57 ± 1 years), including 14 (23%) men and 46 (77%) women, who were admitted to the Department of Rheumatology of the University Hospital of Kazan Federal University during 2017–2018. The study was approved by the Ethical Committee (Reference #7 as of 4 December 2017) of Kazan Federal University and written informed consent was obtained from the patients. The study was performed following the Helsinki Declaration consensus. All data from patients were treated anonymously.

RA was verified based on the diagnostic criteria of the American College of Rheumatology (ACR-2010) and the European League Against Rheumatism (EULAR-2010) [59]. Patients were enrolled in the study if they were at least 18 years old and had been diagnosed with RA based on the criteria of EULAR/ACR 2010 at all stages of the disease progression and with any degree of the disease activity assessed by DAS 28. Patients were excluded from this study if for any reason they took anticoagulants, thrombolytics, or antiplatelet drugs at least 14 days prior to examination. The following patient categories were also excluded: pregnant women and nursing mothers, patients with cancer, active liver disease, bacterial endocarditis, uncontrolled hypertension, thrombocytopenia, anemia, abnormal creatinine clearance test, hemorrhage, and antiphospholipid syndrome.

Patients with RA were treated according to the guidelines of EULAR and ACR. At the time of examination, 23 (38%) patients received methotrexate and sulfasalazine; nine (15%) patients, who were resistant to the basic therapy, were treated with certolizumab pegol (a tumor necrosis factor blocker) and abatacept (an inhibitor of T-cell activation). Thirty-one patients (52%) received glucocorticoids, orally and/or by infusions; intraarticular injection was performed in one patient. The average daily oral dose of glucocorticoid equivalent to prednisolone was 8 mg/day, the average daily dose of glucocorticoid for I.V. administration was 122 mg/day, and the average cumulative dose of glucocorticoids per course was 366 mg. One patient received an injection of diprospan (0.5 mL) in the ankle joint. Thirty-five (58%) patients at the time of examination were on non-steroidal anti-inflammatory drugs, including diclofenac, ibuprofen, and ketorolac. In addition, pentoxifylline was prescribed for 23 (38%) patients with concomitant peripheral circulatory disorders, such as obliterating endarteritis, diabetic angiopathy, Raynaud’s syndrome, and atherosclerotic and discirculatory angiopathy.

The patients with RA were segregated into subgroups based on clinical and laboratory signs of disease severity according to DAS 28 (Disease Activity Score 28). This score includes an assessment of disease activity in 28 joints, also taking into account the erythrocyte sedimentation rate (ESR) and the level of C-reactive protein in the blood. The patients were also divided into four subgroups according to the Steinbrocker Staging System. In addition, patients with RA were allocated into subgroups with a different disease activity according to ESR values, levels of C-reactive protein, absence or presence of antibodies to the cyclic citrullinated peptide (ACCP), and the rheumatoid factor in the blood. The summarized clinical characteristics of the patients included in this study are presented in Table 2.

The control group consisted of 50 healthy subjects 26–77 years old (on average 53 ± 2 years), of which 16 (32%) were men and 34 (68%) women. Healthy volunteers were enrolled in the study provided they did not have a personal or family history of rheumatic arthritis or any other chronic joint diseases or autoimmune pathology. Therefore, the control group matched the group of RA patients by gender- and age-based composition.

### 4.2. Blood Collection and Processing

Blood was collected into vacutainers containing 3.8% trisodium citrate 9:1 by volume and analyzed within 4 h. Sample I of citrated whole blood was used for the clot contraction assay and for platelet isolation. Sample II was centrifuged (1600× *g*, 15 min) to obtain platelet-poor plasma used for blood coagulation tests. For the Thrombodynamics assay, platelet-free plasma was obtained by additional centrifugation of the platelet-poor for 5 min at 10,000× *g* at room temperature. Non-stabilized blood Sample III was collected into a container with a clotting activator silicate and allowed to clot for 30 min at 37 °C, then it was centrifuged for 10 min at 2000× *g* to obtain serum, which was used for biochemical and immunological tests. The blood sample was stabilized with K_3_-ethylenediaminetetraacetic acid (EDTA) and used for hematological tests. Comparative results of routine laboratory tests in the patients and healthy subjects are summarized in Table 3.

### 4.3. Kinetics of Spatially Directed Clot Formation in Blood Plasma (Thrombodynamics Assay)

The Thrombodynamics assay was based on the optical registration of the spatial growth of a fibrin clot in blood plasma upon contact with a surface with immobilized tissue factor that triggers clotting in the adjacent plasma layer. The process of formation and propagation of a fibrin clot was tracked and recorded optically by a CCD camera built into an automated Thrombodynamics Analyzer System (HemaCore Ltd., Moscow, Russia) (Figure 1A). To perform the test, a 200-μL sample of citrated platelet-free plasma was mixed with 200 μg/mL corn trypsin inhibitor to block the contact phase and 20 mM calcium acetate (final concentrations). Then the recalcified plasma sample (120 μL) was transferred to a measuring cuvette, heated to 37 °C, and a plastic activator coated with tissue factor (enclosed in the kit) was immersed. Fibrin growth beginning from one side of the cuvette was registered every 6 s for 30 min (Figure 1A). The serial images were automatically processed by software that calculated the following parameters: (1) lag time, i.e., the time required to start the formation of fibrin from the moment of contact of the plasma with the activating surface; (2) the initial growth rate of the clot, i.e., the median growth rate of the clot calculated between 2 and 6 min after the onset of clot growth; (3) the stationary growth rate of the clot, i.e., the median growth rate of the clot calculated between 15 and 25 min after the onset of clot growth; (4) the size of the fibrin clot after 30 min following the contact of plasma with the activator insert; and (5) clot density, i.e., an optical parameter equal to the intensity of light scattering by the fibrin clot, proportional to the fiber density of the fibrin network (6) the presence and time of formation of spontaneous clots in the plasma volume not adjacent to and not triggered by the tissue-factor coated activating surface.

### 4.4. Optical Registration of the Blood Clot Contraction Kinetics

The kinetics of the contraction of blood clots was recorded using the optical Thrombodynamics Analyzer System (Figure 1B). Citrated blood samples from patients and healthy subjects were activated with 1 U/mL human α-thrombin (Sigma-Aldrich, St. Louis, MO, USA) and 2 mM CaCl_2_ (final concentrations). The activated blood samples (80 μL) were quickly transferred to a 12 × 7 × 1 mm double-spaced transparent plastic cuvette that was pre-coated with a thin layer of 4% vol/vol Triton X-100 in 150 mM NaCl to prevent the clot from sticking to the walls of the chamber without affecting the clot structure and platelet functionality. The transparent cuvette was placed into the 37 °C temperature-controlled chamber of the instrument. The cuvette had two compartments and the experiments were performed simultaneously in duplicate. Images of the clots were taken every 15 s for 20 min to track the changes in the relative clot size based on the light scattering (Figure 1B). The serial images were analyzed computationally to extract the following parameters of clot contraction: (1) extent of contraction calculated as [(*S*_0_ − *S_t_*)/*S_0_*] × 100, where *S*_0_ is the initial clot size and *S_t_* is the final clot size at the 20-min. endpoint; (2) lag time is the time from the addition of thrombin until the clot reaches 95% of its initial size; (3) the average contraction velocity, which is an average of the first derivative of the contraction kinetic curve; and (4) the area under the contraction kinetic curve, roughly corresponding to the amount of mechanical work of clot compression done by the contracting platelets.

### 4.5. Platelet Isolation

Citrated venous blood from RA patients or healthy subjects was spun at 200 g for 10 min to obtain platelet-rich plasma (PRP). Isolated platelets were collected in the void volume after gel filtration of the PRP on Sepharose 2B equilibrated with Tyrode’s buffer (4 mM HEPES, 135 mM NaCl, 2.7 mM KCl, 2.4 mM MgCl_2_, 5.6 mM D-glucose, 3.3 mM NaH_2_PO_4_, 0.35 mg/mL bovine serum albumin, pH 7.4). Cell viability was 97% based on the maintenance of the mitochondrial membrane potential (ΔΨm) determined by flow cytometry using a ΔΨm-sensitive fluorescent dye MitoTracker Deep Red FM (Invitrogen, Carlsbad, CA, USA). Platelet count was performed in a hemocytometer. Platelets were used within 3 h of blood collection.

### 4.6. Flow Cytometry of Resting and Activated Platelets

Platelet functionality was assessed in 27 RA patients and 15 healthy individuals in parallel experiments (one control for one or more RA samples analyzed at a time) by expression of P-selectin (CD62p) and active integrin αIIbβ3 (determined by its fibrinogen-binding capacity) before and after activation with the PAR1-specific thrombin receptor-activating peptide 6 (TRAP-6)(Bachem Americas Inc., Torrance, CA, USA). TRAP-6 was added to isolated platelets at 50 μM and incubated for 3 min at room temperature. Then platelets (200,000 in 50 μL in Tyrode’s buffer) were mixed with 0.045 μg/mL anti-human-CD62p phycoerythrin-labeled murine antibodies (BD Biosciences, San Jose, CA, USA) or 5 μg/mL Alexa Fluor 488-labeled human fibrinogen (ThermoFisher Scientific, Waltham, MA, USA) and incubated for 10 min. After incubation with the labeled ligands, the platelets were analyzed using a FacsCalibur flow cytometer equipped with BD Cell-Quest software (Table 1 and Figure S1). Platelets were gated based on their size and granularity and 5000 platelets were counted in each sample. FlowJo X software was used for data analysis.

### 4.7. Coagulation, Hematological, Biochemical, and Immunological Tests

Blood samples from all RA patients underwent routine cell count, hemostasis, hematological, biochemical, and immunochemical tests. Citrated blood plasma was analyzed using the automated coagulometer system ACL TOP 500 (Beckman Coulter, Brea, CA, USA) for activated partial thromboplastin time (aPTT), prothrombin time (prothrombin ratio), and concentration of fibrinogen (Clauss method). Hematological tests were performed in an automated cell counter system ABX Micros 60 (Horiba, Kyoto, Japan) to retrieve red blood cell, leukocyte, and platelet counts. The following biochemical tests were performed by analyzing serum in the automated analyzing system Cobas Integra 400 plus (Cobas, Basel, Switzerland): total serum protein, total bilirubin, creatinine, glucose, aspartate transaminase, alanine transaminase, uric acid, serum iron, alkaline phosphatase, gamma-glutathione transferase, and cholesterol. Serum antibodies (IgA, IgG, and IgM) and circulating immune complexes were analyzed in the automated system Freedom EVO clinical (TECAN, Crailsheim, Germany). The concentration of anti-dsDNA antibodies, anti-cyclic citrullinated peptide antibodies, and anticardiolipin antibodies were determined using ELISA in the Alegria reader (Orgentec, Mainz, Germany). Serum CH50 complement levels were determined by colorimetry [60] using a KFK-3-01-30MZ photometer (ZOMZ, Sergiev Posad, Moscow, Russia) (Table 3).

### 4.8. Statistical Analysis

Statistical analyses were performed using GraphPad Prism 7 (GraphPad Software, La Jolla, CA, USA). Normality of data distribution was assessed with the Shapiro–Wilk and D’Agostino–Pearson criteria. Pairwise statistical differences were estimated using the Student’s *t*-test (parametric analysis) and Mann-Whitney test (nonparametric analysis). Statistical differences for multiple comparisons were estimated using the Kruskal-Wallis one-way analysis of variance test or two-way ANOVA followed by the Benjamini, Krieger, and Yekutieli test for the false discovery rate. Correlation analysis was performed using the Spearman’s rank correlation coefficient. The level of significance was 95% (*p* < 0.05). The data in the tables are presented as the median and interquartile interval (25th and 75th percentiles) or the mean ± SEM.

## 5. Conclusions

The results show that the parameters of the Thrombodynamics assay and contraction of blood clots in patients with RA are significantly altered compared to healthy donors. In the Thrombodynamics assay, an increase in the spatiotemporal fibrin growth rate, and the size and optical density of a plasma clot in patients with RA indicate pronounced chronic hypercoagulability. The blood clot contraction assay shows a substantial decrease in the extent of clot contraction, median velocity, the area under the kinetic curve (work done by platelets), and a longer lag time in patients with RA compared with the control group. The suppressed contraction of blood clots is due to platelet dysfunction and pathological changes in the composition of the blood caused by systemic inflammation. Platelet dysfunction originated from continuous activation induced by immune inflammatory agents followed by metabolic exhaustion and partial secondary refractoriness, i.e., reduced ability to respond to a physiological stimulus. Hyperfibrinogenemia can also contribute to the impaired contraction of blood clots via increased stiffness, which, in combination with reduced susceptibility to fibrinolysis, has prothrombotic potential. The results obtained indicate the involvement of plasma hemostasis and platelets in the pathogenesis of RA and provide a basis for the predisposition of RA patients to thrombotic complications. Moreover, the results confirm the validity of the Thrombodynamics and clot contraction assays as indicators of the (pro)thrombotic condition caused by systemic inflammation in RA.

## Figures and Tables

**Table 1:** Characterization ^1^ of platelets isolated from the blood of RA patients and control healthy subjects before and after stimulation with the thrombin receptor-activating peptide (TRAP).

Clinical Groups	P-Selectin Expression			Fibrinogen-Binding Capacity		
	Unstimulated Platelets	TRAP-Activated (3-min Activation)	TRAP-Activated (10-min Activation)	Unstimulated Platelets	TRAP-Activated (3-min Activation)	TRAP-Activated (10-min Activation)
Healthy subjects (*n* = 9)	1.4 ± 0.2 ^1^	68.4 ± 3.5 ^1^	87.3 ± 4.7 ^1^	0.8 ± 0.2 ^1^	74.9 ± 5.5 ^1^	75.1 ± 4.7 ^1^
RA patients (*n* = 12)	3.4 ± 0.6 ^1^	38.8 ± 5.9 ^1^ ***	67.8 ± 5.8 ^1^ **	2.6 ± 1.1 ^1^	53.2 ± 6.2 ^1^ **	59.7 ± 6.2 ^1^ *

^1^ Numbers represent the fractions of platelets (%) bearing fluorescently labeled antibodies to P-selectin or labeled fibrinogen. Results are presented as the mean ± SEM. * *p* < 0.05; ** *p* < 0.01; *** *p* < 0.01 between the controls and RA patients (two-way ANOVA and the Benjamini, Krieger and Yekutieli post hoc test for multiple comparisons).

**Table 2:** Clinical characteristics of the RA patients enrolled in this study.

Characteristics		RA Patients (*n* = 60)
Age, years (mean ± SEM)		57 ± 1
Gender	Men	14 (23%)
	Women	46 (77%)
Duration of the disease, years (mean ± SEM)		9 ± 1
Index of disease activity DAS28	Low (2.6–3.2)	18 (30%)
	Medium (3.2–5.1)	21 (35%)
	High (>5.1)	21 (35%)
Main clinical manifestations	Polyarthritis	49 (82%)
	Extra-articular manifestations	8 (13%)
Associated musculoskeletal disorders	Osteoarthrosis	48 (80%)
	Osteoporosis	11 (18%)
Cardiovascular disorders	Hypertonic disease	30 (50%)
	Heart lesions (hypertrophy, coronary heart disease, mitral valve prolapse, dilatation, extrasystole, hydropericardium)	23 (38%)
	Chronic heart failure	26 (43%)
Hematologic disorders	Anemia (Hb < 110 g/L)	23 (38%)
	Thrombocytosis (>420 × 10^9^/L)	8 (13%)
	Hyperfibrinogenemia (>4.5 g/L)	34 (57%)
	Leukocytosis (>9 × 10^9^/L)	4 (6%)
	Leukopenia (<4 × 10^9^/L)	2 (3%)
Renal disorders	Kidney cyst	16 (27%)
	Chronic renal failure	7 (12%)
Gastrointestinal disorders	Liver steatosis	9 (15%)
	Chronic cholecystitis	22 (37%)
	Chronic gastroduodenitis	12 (19%)
	Chronic gastritis	22 (37%)
	Chronic pancreatitis	10 (17%)

**Table 3:** Laboratory parameters in the RA patients compared with the control group.

Parameters (in Parentheses—Reference Values)	RA Patients (*n* = 60)	Control Group (*n* = 50)
Hemostatic parameters		
aPTT (25–36), s	34.3 (31.2; 37.3) **	32.2 (29.7; 34,1)
Prothrombin time (9.4–12.5), s	10.9 (10.4; 11.4) *	11.3 (10.8; 11.9)
Prothrombin index (77–137), %	109 (99; 117) *	99 (92; 110)
Fibrinogen (1.8–4.0), g/L	4.6 (4.1; 5.0) ***	3.8 (3.1; 5.0)
Hematological parameters		
Platelet count (180–320) × 10^9^/L	281 (230; 338) ***	231 (174; 275)
Red blood cells (3.7–4.7) × 10^12^/L	4.3 (3.9; 4.6) ***	4.5 (4.3; 4.9)
Leukocytes (4–9) × 10^9^/L	6.1 (5.0; 8.2) ***	4.9 (4.3; 6.1)
Neutrophils (47–72), %	58 (51; 65)	57 (52; 61)
Lymphocytes (17–48), %	28 (22; 34) *	34 (26; 36)
Monocytes (4–10), %	8.5 (6.4; 10.8) ***	6.2 (5.2; 7.1)
Eosinophils (1–5), %	2.2 (1.3; 3.7)	2.0 (2.0; 4.0)
Erythrocyte sedimentation rate (ESR) (2–20), mm/h	34 (25; 45) ***	7.0 (5.5; 11.5)
Hemoglobin (11.0–16.5), g/dL	11.3 (10.5;12.9) ***	14.3 (12.8; 15.2)
Mean corpuscular volume (MCV) (80–97), fL	84 (74; 90)	86 (83; 89)
Mean corpuscular hemoglobin (MCH) (26.5–33.5), pg	28 (24; 30) ***	32 (31; 34)
Mean corpuscular hemoglobin concentration (MCHC) (31.5–36.0), g/dL	32.5 (31.4; 34.1) ***	35.6 (35.0; 36.2)
Red cell distribution width (RDW) (10–15), %	14.8 (13.4; 16) **	12.6 (12.1; 13.6)
Mean platelet volume (MPV) (6.5–11.0), fL	10.3 (9.2; 10.8) ***	8.2 (7.4; 8.9)
Platelet distribution width (PDW) (10–18), %	12.3 (10.2; 12.9)	12.7 (11.9; 18.4)
Thrombocrit (PCT) (0.12–0.36), %	0.27 (0.23; 0.33) **	0.20 (0.16; 0.24)
Immunological parameters		
IgA (0.8–4.0), mg/mL	1.9 (1.7; 2.6)	2.1 (1.4; 3.3)
IgM (0.5–2.0), mg/mL	2.2 (1.6; 3.3) ***	1.4 (1.3; 1.5)
IgG (5.3–16.5), mg/mL	12.5 (10.5; 17.6) **	9.8 (9.2; 11.6)
Circulating immune complexes (CICs) (0–120), a.u.	99 (84; 146)	95 (86; 101)
Complement CH50 (50–80), hemolytic units	39 (33; 44) ***	56 (52; 61)
Anti-DNA antibody (≤25), IU/mL	4.8 (2.9; 7.5)	2.8 (2.2; 8.1)
Anti-cardiolipin antibody (≤10), IU/mL	2.2 (1.4; 4.0) **	1.2 (0.9; 1.2)
Antibodies to citrullinated peptide (0–5), IU/mL	200 (40; 954) ***	2.0 (1.2; 3.6)
Biochemical parameters		
ALT (5–40), IU/L	13 (9; 20) **	17 (13; 28)
AST (10–36), IU/L	16 (13; 23)	18 (14; 20)
Protein (64–84), g/L	69 (66; 73) ***	73 (70; 76)
Glucose (3.8–6.1), mmol/L	4.9 (4.6; 5.1) *	5.2 (4.7; 5.5)
Creatinine (62–110), μmol/L	53 (44; 65) ***	89 (80; 98)
Bilirubin (3.5–25), μmol/L	6.4 (5.5; 9.0)	8.0 (5.8; 10.0)
Cholesterol (2.2–5.7), μmol/L	4.6 (4.0; 5.4)	4.7 (4.1; 5.0)
Uric acid (202–420), μmol/L	251 (186; 310) ***	355 (284; 399)
Serum iron (10–34.5), μmol/L	9.1 (7.0; 11.9) ***	19 (11; 27)
Alkaline phosphatase (40–130), IU/L	81 (59; 105)	92 (76; 104)
GGT (8–61), IU/L	23 (15; 45)	20 (11; 27)

Results are presented as the median and interquartile range (25th and 75th percentiles). * *p* < 0.05; ** *p* < 0.01; *** *p* < 0.001 (Mann-Whitney U test).

## Supplementary Materials

Supplementary materials can be found at https://www.mdpi.com/1422-0067/21/24/9434/s1.

## Abbreviations

RA	Rheumatoid Arthritis
IL-6	Interleukin-6
*Vi*	Initial Rate of Clot Growth
*Vs*	Stationary Rate of Clot Growth
*D*	Clot Optical Density
RBCs	Red Blood Cells
ESR	Erythrocyte Sedimentation Rate
IQR	Interquartile Range
NSAIDs	Non-Steroidal Anti-Inflammatory Drugs
TRAP	Thrombin Receptor-Activating Peptide
ACCP	Antibodies Against Cyclic Citrullinated Peptide
ACR	American College of Rheumatology
EULAR	European League Against Rheumatism
DAS	Disease Activity Score
EDTA	Ethylenediaminetetraacetic Acid
I.V.	Intravenous
PAR1	Protease-Activated Receptor 1
