# Supplementary material for: Accelerated Spatial Fibrin Growth and Impaired Contraction of Blood Clots in Patients with Rheumatoid Arthritis
Source: Int J Mol Sci. 2020 Dec 11;21(24):9434. doi: 10.3390/ijms21249434 (PMC7764115; doi:10.3390/ijms21249434)
Supplement: Supplementary file 1 [file ijms-21-09434-s001.pdf]

SUPPLEMENTARY MATERIAL

Accelerated Spatial Fibrin Growth and Impaired Contraction of Blood Clots in Patients with Rheumatoid Arthritis

Alina D. Peshkova[1], Tatiana A. Evdokimova[1], Timur B. Sibgatullin[2],
Fazoil I. Ataullakhanov[3], Rustem I. Litvinov[1,4], John W. Weisel[4]

[1]Institute of Fundamental Medicine and Biology and [2]University Hospital, Department of Rheumatology, Kazan Federal University, 420008 Kazan, Russian Federation
[3]Center for Theoretical Problems of Physico-Chemical Pharmacology, Russian Academy of Sciences, 119991 Moscow, Russian Federation
[4]Department of Cell and Developmental Biology, University of Pennsylvania Perelman School of Medicine, 19104 Philadelphia, PA, USA

Supplementary Figure

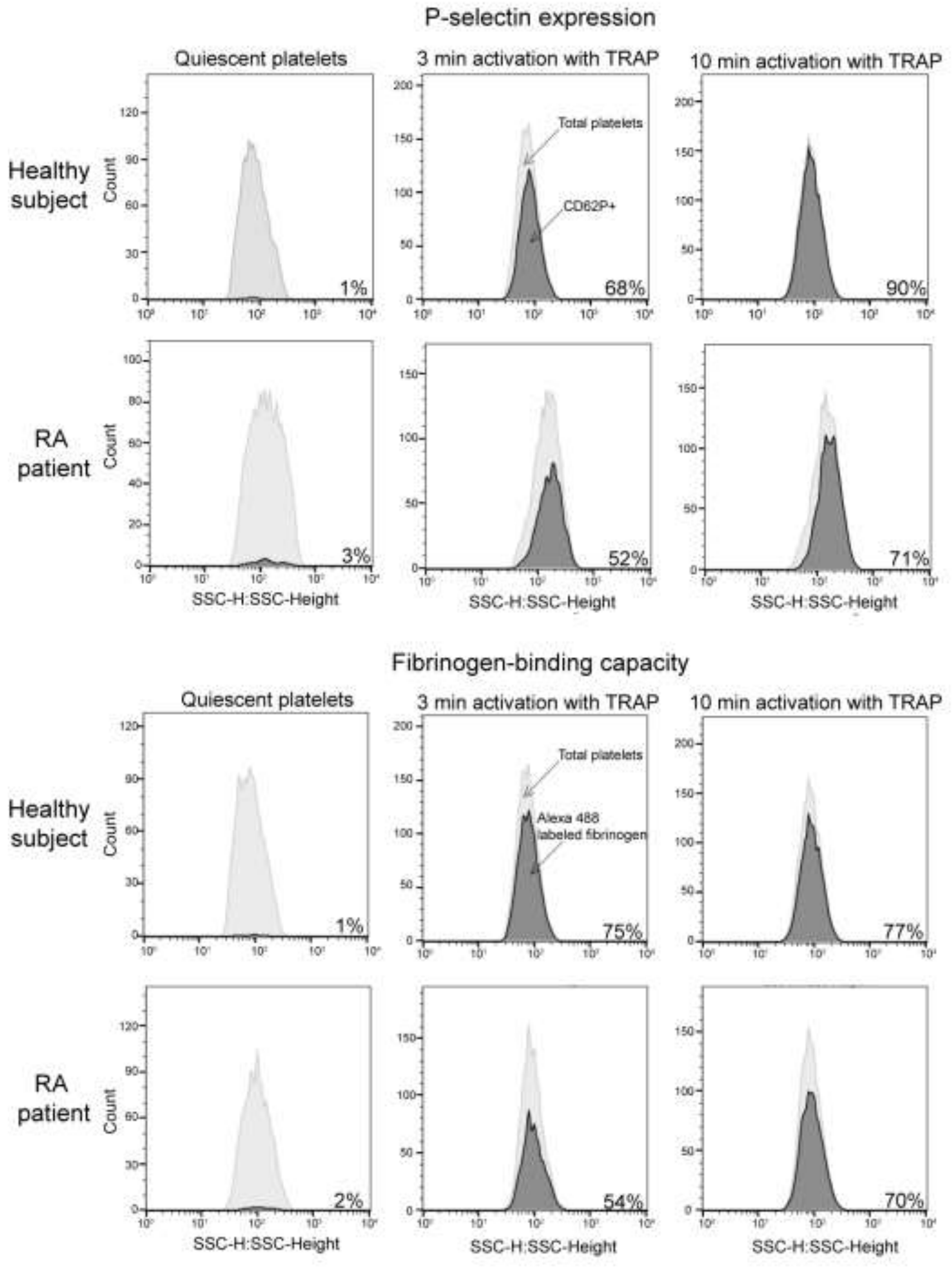

Figure S1. Representative raw data from flow cytometry of platelets isolated from the blood of a healthy donor and an RA patient under various experimental conditions. The platelets were incubated for 3 or 10 minutes with either anti-human CD62 phycoerythrin-labeled antibodies or Alexa fluor 488-labeled human fibrinogen before and after activation with thrombin receptor-activating peptide (TRAP). Each plot represents the peak of counts for fluorescent platelets (dark grey) superimposed on the peak of total platelet counts (5,000 total counts each). Numbers (%) in the lower right corner represent the fraction of fluorescent platelets.

Supplementary Tables

Table S1. Parameters of the Thrombodynamics assay in RA patients compared with the control group of healthy subjects

Parameters	*RA patients (n=60)*	*Control group (n=50)*
Lag-time, min	0.9 (0.8; 1)	0.9 (0.8; 1)
Initial rate of clot growth, μm/min	53.5 (49.8; 57.2)**	50.1 (47.2; 51.2)
Stationary rate of clot growth, μm/min	34.3 (29.6; 38.8)***	28.5 (26.1; 30.5)
Clot size, μm	1262 (1159;1376)***	1128 (1046; 1179)
Clot optical density, a.u.	25398 (22934; 27690)*	21662 (19935; 25784)

Results are presented as a median and interquartile range (25th and 75th percentiles)
*p<0.05; **p<0.01; ***p<0.001 (Mann-Whitney U test)

Table S2. Correlation analysis of the parameters of Thrombodynamics assay and blood clot contraction between each other and other laboratory parameters (Spearman's coefficient)

Correlated parameters	*Parameters of Thrombodynamics assay*					
	Lag time	*Initial rate of clot growth (V_i)*	*Stationary rate of clot growth (V_{st})*	*Clot size*	*Clot optical density (D)*	*Time to formation of spontaneous clots*
Initial rate of clot growth *(V_i)*	-0.214	-	**0.587*** **	**0.776*** **	0.081	0.399
Stationary rate of clot growth *(V_{st})*	-0.125	**0.587*** **	**-**	**0.947*** **	0.024	-0.136
Clot size	-0.169	**0.776*** **	**0.947*** **	-	0.073	0.013
Fibrinogen level	0.177	-0.030	**-0.236***	-0.175	**0.535*** **	0.376
Extent of clot contraction	-0.067	**0.233***	**0.246***	0.210	0.016	-0.090
aPPT	0.168	**-0.384** **	-0.271	-0.272	0.225	0.078
Erythrocyte sedimentation rate	0.205	-0.051	**-0.218***	-0.218	**0.321***	0.262
Anti-DNA antibody level	**0.435** **	-0.039	-0.218	-0.125	0.103	0.162
Anti-cardiolipin antibody level	0.220	**-0.327***	-0.273	-0.273	0.219	-0.542
Monocyte count	0.006	-0.063	-0.015	0.007	0.074	**-0.654** **

Correlated parameters	*Parameters of clot contraction*			
	Extent of clot contraction	*Lag time*	*Area under the curve*	*Average velocity*
Extent of clot contraction	-	**-0.484*** **	**0.843*** **	**0.922*** **
Lag time of clot contraction	**-0.484*** **	-	**-0.645*** **	**-0.482*** **
Area under the kinetic curve	**0.834*** **	**-0.645*** **	-	**0.902*** **
Average velocity of contraction	**0.922*** **	**-0.482*** **	**0.902*** **	-
Platelet count	**0.321***	-0.113	**0.320***	**0.369***
RBC count	**-0.295***	-0.074	-0.163	-0.182
Hemoglobin	**-0.255***	0.067	-0.173	-0.157

*p<0.05; **p<0.01; ***p<0.01

Table S3. Parameters of blood clot contraction in RA patients compared with the control group of healthy subjects

Parameters	*RA patients (n=60)*	*Control group (n=50)*
Extent of clot contraction, %	37 (31; 41)***	49 (45; 53)
Lag time, sec.	255 (180; 293)***	105 (45; 150)
Average velocity, %/sec	0.029 (0.023; 0.034)***	0.039 (0.036; 0.042)
Area under the curve, a.u.	249 (175; 307)***	395 (355; 432)

Results are presented as a median and interquartile range (25th and 75th percentiles).
***p <0.001 (Mann-Whitney U test)

Table S4. Phase kinetic parameters of clot contraction in clots made from the blood of RA patients and control healthy subjects

Rate constants	*RA patients (n=60)*	*Control group (n=50)*
Phase 1, 1/s	0.0001 (0.0001; 0.0379)	0.0166 (0.0001;0.0325)
Phase 2, %/s	0.035 (0.0158; 0.0544)****	0.057 (0.0407; 0.0787)
Phase 3, 1/s	0.002 (0.0002; 0.0035)	0.002 (0.00142; 0.00316)

Results are presented as a median and interquartile range (25th and 75th percentiles).
***p<0.0001 between the values for phase 2

Table S5. Parameters of blood clot contraction in RA patients, taking and not taking non-steroidal anti-inflammatory drugs (NSAIDs)

Parameters	*Patients taking NSAIDs (n=42)*	*Patients not taking NSAIDs (n=18)*
Extent of clot contraction, %	37 (31; 43)	36 (30; 41)
Lag time, sec.	218 (180; 278)	240 (191; 315)
Average velocity, %/sec.	0.031 (0.025; 0.035)	0.029 (0.021; 0.032)
Area under the curve, a.u.	257 (189; 318)	255 (147; 305)

Results are presented as a median and interquartile range (25th and 75th percentiles) (Mann-Whitney U test)
